# mTOR Inhibitor Therapy and Metabolic Consequences: Where Do We Stand?

**DOI:** 10.1155/2018/2640342

**Published:** 2018-06-24

**Authors:** Aleksandra Kezic, Ljiljana Popovic, Katarina Lalic

**Affiliations:** ^1^Faculty of Medicine, University of Belgrade, Dr Subotica 8, 11000 Belgrade, Serbia; ^2^Clinic for Nephrology, Clinical Center of Serbia, Pasterova 2, 11000 Belgrade, Serbia; ^3^Clinic for Endocrinology, Diabetes and Metabolic Diseases, Clinical Center of Serbia, Dr Subotica 13, 11000 Belgrade, Serbia

## Abstract

mTOR (mechanistic target of rapamycin) protein kinase acts as a central integrator of nutrient signaling pathways. Besides the immunosuppressive role after solid organ transplantations or in the treatment of some cancers, another promising role of mTOR inhibitor as an antiaging therapeutic has emerged in the recent years. Acute or intermittent rapamycin treatment has some resemblance to calorie restriction in metabolic effects such as an increased insulin sensitivity. However, the chronic inhibition of mTOR by macrolide rapamycin or other rapalogs has been associated with glucose intolerance and insulin resistance and may even provoke type II diabetes. These metabolic adverse effects limit the use of mTOR inhibitors. Metformin is a widely used drug for the treatment of type 2 diabetes which activates AMP-activated protein kinase (AMPK), acting as calorie restriction mimetic. In addition to the glucose-lowering effect resulting from the decreased hepatic glucose production and increased glucose utilization, metformin induces fatty acid oxidations. Here, we review the recent advances in our understanding of the metabolic consequences regarding glucose metabolism induced by mTOR inhibitors and compare them to the metabolic profile provoked by metformin use. We further suggest metformin use concurrent with rapalogs in order to pharmacologically address the impaired glucose metabolism and prevent the development of new-onset diabetes mellitus after solid organ transplantations induced by the chronic rapalog treatment.

## 1. Introduction

The mammalian target of rapamycin (mTOR) is a cytoplasmic serine/threonine protein kinase that belongs to the phosphoinositide 3-kinase, PI3K-related kinase family, which operates as a central regulator of cell metabolism, growth, proliferation, and survival. It is activated by nutrients (glucose, amino acids, and lipids), growth factors, insulin, and inflammatory cytokines [1, 2]. The mTOR has a unique intracellular signaling position, integrating all those factors, and is a critical regulator of the immune response because it plays a central role in sensing nutrient availability, cytokine/growth factor signaling, and costimulatory factors. Except from the inhibition of interleukin-2-induced T-cell proliferation, mTOR inhibitors induce the development of Treg cells, suppress dendritic cell proliferation and maturation, and play so many complex roles in immune cell cross-talks, including the promotion of proinflammatory cytokine production in some circumstances [3–6].

The increasing use of mTOR inhibitors in recent years, as immunosuppressants both in solid organ transplantation and in the treatment of certain tumors, such as the advanced renal cell carcinoma, also has confronted us with the development of the unwanted effects of this therapy. The development of the adverse effects is primarily a consequence of pleiotropy, a central role for mTOR in a variety of signaling pathways regulating metabolism, growth, and senescence. Among the most common undesirable effects of mTOR inhibitor therapy is metabolic syndrome that implies hyperglycemia with de novo diabetes mellitus (DM) and dyslipidemia.

The retrospective analysis of data from the US Renal Data System (*N* = 20,124 renal transplant patients) has shown that sirolimus was independently associated with an increased risk of new-onset DM [7]. The patients treated with everolimus may develop new-onset diabetes mellitus in up to 32% of cases as a result of hyperglycemia and insulin resistance [8]. The prevalence of hyperlipidemia is significantly higher and occurs in as many as 75% of the patients who are treated with mTOR inhibitors [9, 10].

However, the already known facts that the increased mTOR activity is associated with insulin resistance [11–13] and that the caloric restriction and short-term treatment with rapamycin have led to an increase in insulin sensitivity and glucose uptake [14, 15] suggest a contradictory or dual role of mTOR and mTOR inhibitors. In this review, we will highlight and compare the mechanisms of mTOR inhibitor therapy to the mechanisms of the excessive activation of mTOR leading to metabolic abnormalities. In addition, we will discuss potential therapeutic strategies to mitigate these abnormalities.

## 2. mTOR Signaling Pathways and Pharmacological Inhibition

mTOR is composed of two distinct multiprotein complexes with different cellular functions named mTORC1 and mTORC2 [16]. mTORC1 complex contains five components: mTOR, which is the catalytic subunit; regulatory-associated protein of mTOR (Raptor); mammalian lethal with Sec13 protein8 (mLST8); proline-rich Akt substrate 40 kDa (PRAS40); and DEP domain containing mTOR-interacting protein (Deptor) [17]. Raptor and mLST8 positively regulate mTOR's activity and functions, whereas PRAS40 and Deptor are the negative regulators of the mTORC1 [18–21].

The main inhibitor of mTORC1 is tuberous sclerosis complex 1 (TSC1) and TSC2. Growth factors, nutrients, cytokines, hormones such as insulin, and cellular energy level activate several pathways such as PI3K-Akt and RAS-mitogen-activated protein kinase (MAPK), leading to the inhibition of the TSC1-TSC2 complex [1, 22]. As a consequence, the uninhibited, that is, activated mTORC1, further through S6 kinase 1 (S6K1), 4E-binding protein-1 (4EBP1), cyclin-dependent kinases (CDKs), and the hypoxia-inducible factor 1*α* (HIF1*α*), promotes energy metabolism, protein synthesis and lipogenesis, proliferation, and growth [22]. Actually, the activated mTORC1 via an interaction between Raptor and a TOR signaling (TOS) motif in S6K and 4EBP1 phosphorylates S6K1 and 4EBP1 [23, 24]. The phosphorylated S6K1 then phosphorylates S6 (40S ribosomal protein S6), thereby enhancing the translation of mRNAs. The role of 4EBP1 is to inhibit the initiation of protein translation. It binds and inactivates the eukaryotic translation initiation factor 4E (eIF4E) [25]. When 4EBP1 is phosphorylated by mTORC1, it dissociates from eIF4E, enabling the increased translation of mRNAs and G1-to-S phase transition [25, 26]. mTORC1 also promotes growth by negatively regulating autophagy, which is the central degradative process in cells, but it is beyond the scope of this article [27].

**Figure 1:**
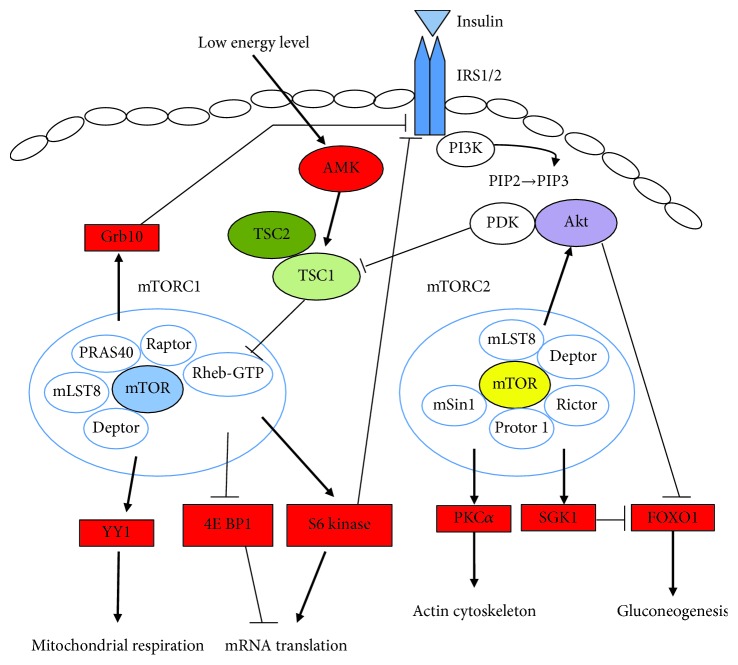
mTOR signaling pathways. IRS 1/2: insulin receptor substrate protein-1/2; PI3K: phosphoinositide 3-kinase; AKT: protein kinase B; Grb 10: growth factor receptor-bound protein 10; AMPK: adenosine monophosphate-activated protein kinase; TSC1: tuberous sclerosis complex 1; TSC2: tuberous sclerosis complex 2; mTORC1: mTOR complex 1; mTORC2: mTOR complex 2; PDK: phosphoinositide-dependent protein kinase 1.

The PI3K/Akt and mTOR signaling are closely interconnected. The binding of growth factors to insulin-like growth factor receptor (IGFR), platelet-derived growth factor receptor (PDGFR), or epidermal growth factor receptor (EGFR) generates downstream signal, which activates the PI3K/Akt pathway. When insulin binds to its cell surface receptor, the recruitment of insulin receptor substrate 1 (IRS) is promoted with the activation of PI3K and the production of phosphatidylinositol (3,4,5)-trisphosphate (PIP3) [2] (Figure 1). PIP3 binds to Akt and then engages this kinase to the cell membrane, to be activated by phosphorylation by PDK1 [28]. Activated Akt phosphorylates several downstream substrates, including TSC1/TSC2 complex, thereby activating mTORC1 and downstream effectors of mTORC1 [29, 30]. The upstream IRS pathway is negatively regulated by the mTOR-S6K1 pathway through a direct phosphorylation on specific residues [31, 32]. This increased degradation of IRS1, caused by hyperphosphorylation on serine/threonine residues, can lead to insulin resistance associated with the mTOR overactivation.

Compared to mTORC1, much less is known about the upstream activators of the mTORC2 pathway. mTORC2 responds to the growth factors such as insulin, via direct associations to ribosome in a PI3K-dependent fashion [33]. mTORC2 directly activates Akt by phosphorylating its hydrophobic motif (Ser473) and SGK1, a kinase controlling ion transport and growth [1, 34]. The loss of mTORC2 does not prevent phosphorylation of some Akt targets such as TSC2 but completely abolishes the activity of SGK1 [34, 35]. Thus, PI3K/Akt, in addition to the activation of mTORC2 by promoting its association with ribosomes, also controls the mTORC1 activation through the Akt-dependent TSC1/TSC2 inhibition [36]. Except from Akt and SGK1, PKC-*α* is another kinase activated by mTORC2, which regulates cell shape by affecting the actin cytoskeleton [37].

Originally, it was thought that acute treatment with rapamycin in contrast to the mTORC1 inhibition does not perturb mTORC2 signaling, but recent data confirm that there is a cell-type specificity to the rapamycin sensitivity of mTORC2 assembly [38]. Anyway, although mTORC2 is less responsive to rapamycin and rapalogs, a prolonged exposure to these compounds leads to a suppressed mTORC2 assembly, with a consequent inhibition of Akt signaling [39]. At the same time, rapalogs therapy results in a reduced or modified efficacy, due to the existence of numerous negative feedback loops in the mTOR pathway. The direct phosphorylation of IRS1 by the mTOR-S6K1 pathway, which promotes IRS1 degradation and PI3K/Akt downregulation, has already been mentioned [31, 32, 40]. That is why rapalogs lead to a decrease in negative feedback of the mTOR-S6K1 pathway on IRS pathway, thereby increasing the growth factor and Akt signaling with a decreased apoptotic potential. This is one of the reasons for insufficient antitumor activity of the mTOR inhibitors.

However, regardless of the association of mTOR overactivation and insulin resistance, rapalogs may also cause insulin resistance and hyperglycemia. In order to explain this phenomenon, it is necessary to look at the effects of mTOR inhibition in several organs, in the first place including the pancreas and the liver.

## 3. Metabolic Consequences of Overactivated mTOR

The postprandial increase of glucose and insulin activates mTOR and consequently protein kinase B (Akt) through mTORC2. The activation of Akt leads to glucose uptake by an increased GLUT4 translocation to the membrane in adipocytes [41]. The GSK-3 phosphorylation and deactivation by Akt decrease the rate of phosphorylation of glycogen synthase and increase the glycogen synthase activity and the accumulation of glycogen, most importantly in the liver and muscles [42]. Additionally, Akt controls glucose homeostasis by phosphorylating and inhibiting FOXO1, a transcription factor that regulates gluconeogenesis [43]. In addition, mTORC2 promotes glycogen synthesis and decreases gluconeogenesis in the liver [44].

As we have already mentioned, it is important to emphasize that both nutrients and insulin activate mTOR, but the overactivated mTOR further causes insulin resistance by at least two mechanisms [13, 32, 45]. S6K1 activated by mTORC1 causes the phosphorylation and degradation of insulin receptor substrate 1/2, thereby impairing insulin signaling. By affecting the growth factor receptor-bound protein 10, mTORC1 may also cause insulin resistance. The deletion of S6K1 is sufficient to improve insulin sensitivity in mice and in fat-fed rodents, while the activated mTOR pathway leads to an impaired insulin signaling and insulin resistance [46, 47]. In humans, the infusion of amino acids activates the mTOR/S6K1 pathway and consequently causes insulin resistance in skeletal muscles [45].

Thus, the overactivation of mTOR in the liver, muscles, adipose tissues, and pancreas leads to insulin resistance. Initially, mTORC1 stimulates *β*-cell functions causing an increased insulin secretion and the expansion and hypertrophy of *β* cells. The mTORC2-Akt axis positively affects *β*-cell mass by promoting proliferation and survival [27]. In further course of the chronic mTOR stimulation, mTOR renders *β*-cells resistant to IGF-1 and insulin, fostering cell death [48, 49]. It means that the overactivated mTORC1 in pancreas *β*-cells causes an increased insulin secretion to compensate for insulin resistance, but eventually, it leads to *β*-cell failure.

The mTOR activity affects lipid metabolism, too. Signaling promotes lipogenesis in the liver. Through sterol regulatory element-binding protein (SREBP), mTOR promotes lipogenesis in the liver [50]. The insulin-stimulated mTORC1 enhances lipogenesis and lipid storage, while it inhibits lipolysis, *β*-oxidation, and ketogenesis. The activated mTORC1 has an impact on three lipases: adipose triglyceride lipase (ATGL), hormone-sensitive lipase (HSL), and lipoprotein lipase (LPL) [51]. In adipocytes, ATGL catalyzes the lipolysis of triacylglycerol to diacylglycerol, and then HSL converts diacylglycerol to monoacylglycerol. mTORC1 reduces the HSL activity and decreases the activity of extracellular LPL, which is important for lipoprotein uptake in tissues. The mTORC1 activation reduces ketone body production by inhibiting PPAR-*α* activity in the liver [27].

By coordinating various levels of the gene expression, mTORC1controls mitochondrial mass and functions. The loss of mTORC1 in the muscle of mice reduces oxidative function and muscle mass leading to an early death [52]. The loss of mTORC1 or rapamycin treatment reduces peroxisome proliferator-activated receptor coactivator 1-alpha (PGC-1*α*) expression and inhibits the complex of PGC-1*α* with the transcription factor yin-yang 1 YY1 [53]. Rapamycin decreases the gene expression of PGC-1alpha, oestrogen-related receptor alpha, and nuclear respiratory factors, which are mitochondrial transcriptional regulators, resulting in a decrease in mitochondrial gene expression and oxygen consumption. YY1 regulates mitochondrial gene expression and is a common target of mTOR and PGC-1alpha. The inhibition of mTOR results in a failure of YY1 to interact and has coactivated by PGC-1alpha, thereby depressing mitochondrial oxidative function [53].

Ultimately, insulin resistance due to elevated mTOR activity, characterized by increased hepatic gluconeogenesis, reduced glucose uptake by muscles, and pancreatic *β*-cell apoptosis, leads to type II diabetes. Taking into consideration that insulin resistance and associated complications such as retinopathy, neuropathy, and nephropathy can precede the diagnosis of type II diabetes raises the question of the possibility for the prevention of diabetic complications using pharmacological inhibition of the mTOR pathway.

## 4. Glucose Intolerance Induced by mTOR Inhibitors

It is obvious that mTOR has multiple roles in metabolism and, when overactivated by nutrient overload and obesity, participates in causing glucose intolerance and insulin resistance. Calorie restriction, which means a reduction in caloric intake, while maintaining adequate nutrition, improves glucose tolerance and insulin sensitivity and extends lifespan [54, 55]. Given the assumption that rapamycin is a starvation mimetic, its role has been suggested in reversing insulin resistance. The acute treatment with rapamycin (single injection) increases insulin sensitivity and glucose uptake [14, 56]. In healthy volunteers, a single dose of rapamycin as a pretreatment abrogates nutrient-induced insulin resistance [57]. In contrast to the results of acute or intermittent rapamycin treatment, the chronic treatment with rapamycin impairs glucose homeostasis. Paradoxically, the chronic rapamycin treatment leads to glucose intolerance in both animals and humans [7, 58, 59]. Although chronic rapamycin treatment reduces fat content, it also promotes insulin resistance, glucose intolerance, and gluconeogenesis in the liver. Despite the improved insulin signaling in the liver of rapamycin-treated rats, which came out from the blockade of the mTOR/S6K1 negative feedback loop, the induction of gluconeogenic pathway in the liver potentiates glucose intolerance [59, 60]. Although white adipose tissue and skeletal muscles take up glucose normally in response to continuous insulin stimulation during the chronic rapamycin treatment, hepatic insulin resistance is a major contributor to the impaired glucose homeostasis [59]. It has been shown that the insulin-mediated suppression of hepatic gluconeogenesis is directly mediated by rapamycin-induced mTORC2 disruption [59]. Except from the mTORC2 inhibition, the chronic rapamycin treatment contributes to insulin resistance, due to inability to activate fatty acid *β*-oxidation and ketogenesis, leading to an imbalance in lipid metabolism [61]. Additionally, a prolonged rapamycin treatment leads to a decreased *β*-cell viability and decreased insulin secretion, probably via the inhibition of mTORC2 [62, 63]. This increased *β*-cell toxicity induced by the chronic mTOR inhibitor treatment might be a bridge leading to the development of new onset of diabetes mellitus after solid organ transplantations, imposing the need of the development of strategies to avoid this adverse effect.

## 5. The Role of Metformin in the Reversal of Insulin Resistance Induced by mTOR Inhibitors

The clinical significance of insulin resistance is associated with coronary artery disease and ischemic stroke [64, 65]. Metformin, a widely prescribed antidiabetes drug, is a biguanide and represents the first line of the treatment for type II diabetes mellitus [66]. It not only decreases hyperglycemia primarily by lowering hepatic gluconeogenesis but also increases insulin sensitivity and lowers blood lipid level [67]. However, in addition to the treatment of type II diabetes mellitus, metformin has shown its beneficial effect in aging-related diseases such as cancer and cardiovascular diseases [68–70]. In all of these aging-related conditions, metformin has achieved effects similar to the effects of rapamycin therapy. Several epidemiological studies have confirmed that the treatment of diabetes type II with metformin was associated with a reduced cancer incidence and cancer-related death [68, 71–73]. Different animal experimental models have as well shown varying anticancer and prolongevity effects depending on dosage, sex, and age at the onset of metformin treatment [74–76].

The molecular mechanisms of metformin are only partially understood. The multiple mechanisms of action have been studied, suggesting inhibition of the mitochondrial respiratory chain (complex I) as the primary mode of action. [77, 78]. As a result, a decrease in cellular energy status with an increased cellular AMP : ATP ratio activates AMP-activated protein kinase (AMPK), which inhibits mTORC1 signaling in the liver, the primary site of metformin action, with different downstream effects [78–80]. At a lower dosage, metformin requires AMPK and the TSC to inhibit mTORC1, whereas at higher dosage, this effect is AMPK and TSC independent [80]. Anyway, metformin decreases a hepatic protein synthesis through a mechanism implicating inhibitory effect on mTORC1. By inducing the phosphorylation of GLUT4 enhancer factor, metformin enhances the peripheral glucose uptake, thereby increasing insulin sensitivity. Additionally, metformin decreases an insulin-induced suppression of fatty acid oxidation [81]. The effect appears to be attributable to a stimulation of AMPK and the reduction of malonyl-CoA content in the muscles.

All these metabolic effects are almost identical to the effects of hunger, that is, dietary restriction. It has been shown that metformin-treated mice had a transcriptional profile resembling mice subjecting to dietary restriction [82]. Although both rapamycin and dietary restriction inhibit lipogenesis and activate lipolysis with consequent increased serum levels of nonesterified fatty acids, in contrast to dietary restriction, rapamycin does not activate *β*-oxidation [61]. Considering that the two main characteristics of metabolic disorder caused by rapamycin are the stimulation of gluconeogenesis in the liver and the decrease of *β*-oxidation, metformin is imposed as a potential solution. Since decreased fatty acid oxidation is associated with the development of insulin resistance, the metformin-induced fatty acid oxidation might contribute to the increase of insulin sensitivity. The addition of metformin to chronic rapamycin treatment may provide a therapeutic approach to treat insulin resistance and dyslipidemia. Most of the literature discusses the combined use of metformin and rapamycin for the purpose of treating aging and aging-related diseases. Another option suggested for prevention, that is, treat metabolic disorder caused by rapamycin, is an intermittent application of rapamycin, taking into account the fact that after the cessation of rapamycin therapy, insulin resistance and glucose intolerance are reversible. It is clear that this approach cannot be used in patients treated with immunosuppressive therapy to prevent transplant rejection or in patients who take mTOR inhibitors as an anticancer treatment, where therapy with mTOR inhibitors must be continuous.

The so-far conducted study summarized rapamycin effects on mTORC1 and mTORC2, pointing to the fact that a long-term treatment with rapamycin in addition to mTORC1 also disrupts mTORC2, thereby causing *β*-cell toxicity and insulin resistance [62, 83]. This effect of mTORC2 inhibition was confirmed in vivo in multiple tissues, including the liver, white adipose tissue, and skeletal muscle [59]. Given the assumption that the immunosuppressive effects of rapamycin are mediated predominantly via mTORC1, one may suppose that the mTORC1-specific inhibitors would achieve the same immunosuppressive effects as rapamycin, but without any mTORC2-mediated toxicity. This assumption might be operating when mTOR inhibitors are used as antiaging therapeutics because mTORC1 inhibition would achieve the desired effects by avoiding metabolic disorders caused by mTORC2 inhibition [84].

Would it be so if the mTORC1-specific inhibitors were used as immunosuppressive drugs? It seems that important immunosuppressive effects of mTOR inhibitor therapy are mediated by the inhibition of both mTORC1 and mTORC2. In addition to the inhibition of T-cell proliferation and blockade of dendritic cell maturation, one of the hallmarks of the immunoregulatory properties of mTOR inhibitors is the development of Tregs whose differentiation and expansion are suppressed by mTORC2 activity [85, 86]. This means that the specific mTORC1 inhibition in the cells belonging to the immune system without mTORC2 disruption may lead to an insufficient immunosuppression.

There are some indications that other rapalogs, such as everolimus and temsirolimus, achieve a lower degree of mTORC2 inhibition and thus a lower degree of insulin resistance, but this still needs to be confirmed in other studies [87].

Anyway, the necessity of the constant use of mTOR inhibitors after solid organ transplantations, such as kidney transplantation, prevents the regimen of intermittent application of rapamycin or the use of rapamycin in smaller doses. In an attempt to solve this problem, that is, to prevent insulin resistance and new-onset diabetes after a kidney transplantation, the combined therapy of rapamycin and metformin has been suggested [88, 89]. By inducing AMPK at clinically relevant doses, metformin inhibits mTORC1, helping to reduce the dose of rapalogs and associated adverse metabolic effects. If the patients with kidney transplants have GFR > 60 ml/min, metformin may be prescribed for the treatment of preexisting type 2 diabetes mellitus or new-onset diabetes mellitus [90].

## 6. Conclusion

We are trying to show that metformin use is also possible in order to prevent the onset of diabetes mellitus after a kidney transplantation. So far, no studies have been carried out to investigate the role of metformin in the prevention of new-onset diabetes mellitus after a transplantation. The future research can result in clinical guidelines, which will allow us to better counteract rapalog-mediated adverse effects.
